# Validation of the Bipolar Disorder Etiology Scale Based on Psychological Behaviorism Theory and Factors Related to the Onset of Bipolar Disorder

**DOI:** 10.1371/journal.pone.0116265

**Published:** 2014-12-30

**Authors:** Jae Woo Park, Kee Hwan Park

**Affiliations:** 1 Department of General Counseling, Korea Counseling Graduate School, Seoul, South Korea; 2 Department of Psychology, The Catholic University of Korea, College of Social Science, Bucheon, South Korea; Catholic University of Sacred Heart of Rome, Italy

## Abstract

**Objectives:**

The aim of this study was to identify psychosocial factors related to the onset of bipolar I disorder (BD). To do so, the Bipolar Disorder Etiology Scale (BDES), based on psychological behaviorism, was developed and validated. Using the BDES, common factors related to both major depressive disorder (MDD) and BD and specific factors related only to BD were investigated.

**Method:**

The BDES, which measures 17 factors based on psychological behaviorism hypotheses, was developed and validated. This scale was administered to 113 non-clinical control subjects, 30 subjects with MDD, and 32 people with BD. ANOVA and post hoc analyses were conducted. Subscales on which MDD and BD groups scored higher than controls were classified as common factors, while those on which the BD group scored higher than MDD and control groups were classified as specific factors.

**Results:**

The BDES has acceptable reliability and validity. Twelve common factors influence both MDD and BD and one specific factor influences only BD. Common factors include the following: learning grandiose self-labeling, learning dangerous behavior, reinforcing impulsive behavior, exposure to irritability, punishment of negative emotional expression, lack of support, sleep problems, antidepressant problems, positive arousal to threat, lack of social skills, and pursuit of short-term pleasure. The specific factor is manic emotional response.

**Conclusions:**

Manic emotional response was identified as a specific factor related to the onset of BD, while parents’ grandiose labeling is a candidate for a specific factor. Many factors are related to the onset of both MDD and BD.

## Introduction

Bipolar I disorder causes severe psychological and financial strain on the patients, their family members, and the public health care administration system. In addition, it causes substantial disability. It has been reported that patients with bipolar I disorder show reduced task performance at work [1]. They are also at a high risk of suicide, with 50% attempting suicide and 6% to 20% committing suicide [2].

As such, the impact of bipolar disorder (BD) is serious. However, there seems to be a lack of well developed specific psychotherapeutic interventions based on etiological factors of the disorder. The lack of research on specific psychosocial etiological factors can be attributed to the fact that genetics have a large influence on the onset of the disorder and pharmacotherapy is the first line of treatment. For schizophrenia (SPR), however, the level of expressed emotion influences the course of the disorder [3]. A high level of expressed emotion is a more powerful predictor of relapse of a family member’s SPR than is taking a neuroleptic medication [4], and the nine-month relapse rates were significantly greater among SPR patients with high expressed emotion family members [5]. According to another research, high criticism, which consists of expressed emotion, is significantly associated with relapse rate, relapse interval, and relapse symptom severity [6]. Further, some of these findings have been proved to be cross-culturally consistent [7]. Based on these studies, it may be suggested that individuals with SPR and their family members can receive intervention from mental health experts to lower their level of expressed emotion, so as to lower the relapse rate. However, despite a few studies on the psychosocial etiological factors of BD, these factors have not been studied sufficiently. It has been reported that a lack of social support and high level of social strain are related to the inter-episode residual mood symptoms and irregular circadian rhythm of BD [8]. Childhood sexual abuse is another factor found to be associated with the severity of BD. Further, it is related to suicidal behavior, substance abuse, and psychotic symptoms of BD [9]. Childhood trauma is also associated with BD and its clinical expression [10]. Indeed, although there are some findings on the psychosocial etiological factors of BD, more researches are needed to clarify the characteristics of trauma and abuse. Studies must seek answers to the following question: “What kind of trauma, or which characteristics of trauma and abuse are associated with bipolar I disorder?” Expressed emotion is a more specific concept than lack of social support, social strain, trauma, or abuse; thus, more specific interventions can be used. Generally speaking, if more specific psychosocial factors are identified, more specific interventions can be designed and used. However, as for BD, this is not the case.

Although medication is the primary therapy for BD, cognitive behavior therapy (CBT) is an additive therapy that aims to reduce depressive and manic symptoms during the depressive episode and inter-episode periods. In CBT, the underlying irrational and dysfunctional beliefs must be explored and restructured to ameliorate the depressive and manic symptoms. Group intervention based on CBT seems to reduce relapse rate and augment the effect of mood stabilizers used to reduce depressive symptoms [11]. However, there is a lack of psychotherapy interventions specific to the psychosocial factors influencing the onset of BD. In this context, the psychological behaviorism theory and psychometric validation methodology may provide clues to this problem. If there are “various and very specific” hypotheses on the etiology of BD, and some of the hypotheses are proved to be true, the findings can be used to modify and refine the intervention of psychotherapy.

Riedel, Heiby, and Kopetskie [12] suggested fifteen hypotheses related to the etiology of bipolar I disorder, based on the psychological behaviorism theory [13]–[15]. Based on psychological behaviorism, fifteen hypotheses were presented to explain the etiological factors of bipolar I disorder. After discussion among the authors of this study and an original author of the psychological behaviorism theory of bipolar disorder, two hypotheses were added to explain irritability symptoms. The aim of the present study was to develop a psychometric assessment scale based on those hypotheses, and to validate it. Further, it aimed to identify the common factors that affect both major depressive disorder and BD, and specific factors that only affect BD. These seventeen hypotheses were adopted with the permission of the original authors and are presented in Table 1.

**Table 1:** Hypotheses Regarding the Etiology of BD Based on Psychological Behaviorism Theory.

Factors	Sub-Factors	Hypotheses
**Past Psychosocial** **Factors**	1. Learning risky activities	Bipolar individuals have had developmental histories in which significant others modeled and shaped risky types of activities, such as gambling, crime, or substance abuse.
	2. Reinforcing impulsivity	Significant others punished a cautious or realistic attitude towards the future and modeled and reinforced impulsivity and, perhaps, denial or disregard of consequences.
**S1-1: Elation**	3. Grandiose self- labeling	Bipolar individuals learned to use grandiose self-labeling and denial as ways to cope with stress as well as to enhance feelings of elation and confidence in response to superficial successes.
**Past Psychosocial** **Factors**	4. Exposure to irritability	Bipolar individuals were exposed to family members’ irritability.
**S1-2: Irritability**	5. Punishment of negative emotions	Bipolar individuals used to be punished for expressing negative emotions including anger.
**Concurrent Psychosocial** **Factors**	6. Euphoria-triggering experience	Manic episodes involving euphoria may be triggered by pleasant events.
	7. Lack of social support	Bipolar individuals may lack adequate social support systems that involve significant others who model and reinforce euthymic emotions, veridical self-labeling and cautionary statements, and safe behaviors.
**S2**	8. Reinforcement for elevated emotions	Significant others may provide reinforcement for elevated emotions, grandiose self-labeling, and risky behavior.
**Transient Concurrent** **Organic Factors**	9. Sleep problems	Medical problems, such as sleep deprivation, may precipitate the onset of bipolar symptoms.
**O3**	10. Antidepressant problems	Medical treatment, such as antidepressant medication, may be iatrogenic.
**e-m BBR**	11. Manic emotional response	Bipolar emotional responses to certain stimulus situations are excessive.
	12. Positive arousal to threat	Bipolar individuals respond with positive emotional arousal to situations that others would find threatening or anxiety provoking.
**s-m BBR**	13. Manipulative Skill	Bipolar individuals have above-average skills for engaging in risky types of activities, which produce short-term reinforcement and long-term punishment. These may include persuasive conversational techniques or social manipulativeness.
	14. Lack of social skills	Bipolar individuals have a deficit in social skills for maintaining adequate social support networks.
**l-c BBR**	15. Short-term pleasure seeking	Bipolar individuals have a deficit in estimating long-term negative consequences.
	16. Denial of negative emotions	Bipolar individuals tend to employ grandiose self-labeling and denial to elevate mood.
	17. Lack of problem-solving skills	Bipolar individuals have poor cognitive problem-solving skills.

Adopted with permission.

## Methods

First, the Bipolar Disorder Etiology Scale (BDES) based on psychological behaviorism was developed and validated. Then, the scale was administered to a non-clinical control group, individuals with major depressive disorder (MDD), and individuals with bipolar I disorder (BD) to identify the common factors related to both MDD and bipolar I disorder and specific factors related only to bipolar I disorder. The Institutional Review Board of Korea Counseling Graduate University approved the study.

### Subjects

The subjects in this study included a non-clinical control group, a MDD group, and a bipolar I disorder group. All of them provided their written informed consent to participate in this study. The non-clinical control group consisted of 113 healthy subjects with no history of a psychiatric illness. The MDD and bipolar I disorder groups were recruited using the Structured Clinical Interview for DSM-IV Axis I Disorders (SCID). Individuals with dysthymia, double depression, or bipolar II disorder were excluded. The sample included 30 participants with MDD and 32 with bipolar I disorder. There were no significant differences between the three groups in terms of age [*F(2,172) = 1.278, p = .281*), education level [*F(2, 123) = .408, p = .666*], or gender [*χ^2^(2) = 3.516, p = .172*)].

### Measurement

#### Bipolar Disorder Etiology Scale based on psychological behaviorism

For each of the sub-factors associated with the 17 hypotheses, five items were developed in Korean. The first draft of the scale was translated into English and revised by Dr. Heiby. Then, it was re-translated into Korean, resulting in an 85-item scale. The scale has been presented in the S1 Table. The subscales of the BDES, and its matched item numbers, have also been presented in the S2 Table. The BDES was administered to all the subjects.

#### Visual Analog Scales for the hypotheses

Since there are no similar scales available to verify construct validity, 17 visual analog scales (VAS) were constructed reflecting each of the 17 hypotheses respectively. To make the data coding process easier, 10 cm bars were replaced by 10-point Likert scales. The VAS was administered to all the subjects. This scale has also been presented in the S3 Table.

### Data analysis

First, to verify the reliability of the scale, internal consistency coefficients for the 17 subscales were calculated. Second, correlational analyses were conducted between the 17 subscales and the 17 VAS scores, respectively, to verify construct validity. Third, a dummy variable was created to distinguish between the non-clinical group (0) and the bipolar I disorder group (1). Then, nonparametric correlational analyses were conducted between the dummy variable and the 17 subscales.

To identify common and specific factors, ANOVAs were conducted with the group variable as the independent variable and the subscale scores as the dependent variable. Scheffe’s tests were used for post hoc analyses.

## Results

### Reliability and validity of the Bipolar Disorder Etiology Scale based on psychological behaviorism

A total of 175 subjects participated in this study, including 113 non-clinical controls, 30 MDD patients, and 32 BD patients. The mean age of the subjects was 33.06. Thirty four percent of participants were male, and 66% were female. Age and gender distributions by group are presented in Table 2. Internal consistency estimates for the 17 subscales ranged from.51 to.90, suggesting that the subscales met the minimum requirements with reference to reliability. Correlation coefficients between subscale scores and the corresponding visual analog scale varied from.19 to.81 (*p<*.05), with the exception of the grandiose self-labeling subscale (*r = .14, NS*). Three out of the 16 correlation coefficients had weak positive correlation coefficients and interestingly, there were no group differences with reference to these three subscales. The three subscales in question were as follows: euphoria-triggering experience, reinforcement for elevated emotion, and denial of negative emotions. The absence of group differences may have been due to the lack of validity of the subscales, because the subscales did not measure significant factors. The revision of these subscales may lead to different findings. Thus, the revision of these subscales must be considered in follow-up studies. It seems that the rest of the thirteen subscales had acceptable validity.

**Table 2:** Demographic Variables.

		Non-clinical	MDD	Bipolar	Total
**Age: M (SD)**		32.48 (11.16)	35.87 (10.19)	32.47 (8.56)	33.06 (10.59)
**N**	**Male**	34	12	15	61
	**Female**	79	18	17	114

Correlation coefficients between subscale scores and diagnostic groups varied from.11 to.43 (*p<*.05), with the exception of the euphoria-triggering experience (r = –.05, NS) and manipulative skill (r = .09, NS) subscales. These results suggest that the scale has an acceptable level of construct validity. The results are presented in Table 3.

**Table 3:** Reliability and Validity of the BDES, with Common and Specific Factors.

							N	M	B			Post	
	Sub-factors	Reliability^1^	Validity^2^		Validity^3^		M (SD)	M (SD)	M (SD)	*F*		hoc	4
**S1-1**	1. Learningrisky activities	.64	.34	**	.42	**	6.36 (2.55)	8.67 (3.54)	9.53 (4.07)	16.78	**	B = D>N	C
	2. Reinforcingimpulsivity	.72	.43	**	.56	**	9.58 (3.91)	14.77 (5.14)	15.88 (4.66)	36.77	**	B = D>N	C
	3. Grandioseself-labeling	.54	.18	*	.14		17.04 (3.82)	18.17 (4.41)	19.03 (4.12)	3.48	*	B>N B = DD = N	C
**S1-2**	4. Exposure toirritability	.90	.36	**	.76	**	10.84 (6.17)	21.80 (8.93)	20.06 (8.35)	40.31	**	B = D>N	C
	5. Punishment ofnegative emotions	.87	.31	**	.74	**	12.77 (5.41)	19.00 (6.55)	18.66 (6.38)	21.85	**	B = D>N	C
**S2**	6. Euphoria-triggeringexperience	.65	–.05		.22	**	12.65 (4.12)	12.41 (4.59)	12.09 (4.30)	0.23		B = D = N	
	7. Lack of socialsupport	.85	.32	**	.58	**	9.59 (4.11)	16.60 (6.73)	15.00 (7.21)	27.97	**	B = D>N	C
	8. Reinforcement forelevated emotions	.78	–.15	*	.19	*	16.11 (5.12)	14.73 (5.69)	14.06 (4.98)	2.32		B = D = N	
**O3**	9. Sleep problems	.77	.22	**	.60	**	13.91 (4.75)	19.27 (5.64)	17.91 (6.40)	16.17	**	B = D>N	C
	10. Antidepressantproblems	.64	.76	**	.58	**	5.55 (1.93)	18.63 (12.42)	17.94 (6.52)	89.79	**	B = D>N	C
**e-mB**	11. Manic emotionalresponse	.82	.33	**	.48	**	10.69 (4.86)	12.90 (5.01)	16.50 (6.25)	16.00	**	B>D = N	s
	12. Positive arousalto threat	.51	.27	**	.50	**	8.46 (2.54)	10.27 (3.23)	11.31 (4.06)	13.12	**	B = D>N	C
**s-mB**	13. Manipulative skill	.70	.09		.35	**	9.20 (3.76)	9.81 (2.58)	10.29 (4.58)	1.09		B = D = N	
	14. Lack ofsocial skills	.83	.33	**	.47	**	14.06 (5.05)	20.67 (4.45)	19.56 (5.38)	29.35	**	B = D>N	C
**l-cB**	15. Short-termpleasure seeking	.77	.21	**	.47	**	10.30 (3.90)	13.03 (5.49)	13.25 (5.06)	8.27	**	B = D>N	C
	16. Denial ofnegative emotions	.61	.11	*	.27	**	15.21 (4.67)	14.47 (4.32)	17.59 (7.50)	3.30	*	B = D = N	
	17. Lack ofproblem-solving skills	.88	.28	**	.56	**	14.03 (4.01)	18.00 (5.51)	18.28 (6.36)	14.88	**	B = D>N	C

1 Reliability: internal consistency, Cronbach’s alpha.

2 Validity: nonparametric correlations between subscale scores and diagnostic dummy code.

3 Validity: correlations between subscale scores and visual analog scales.

4 Factors: C = common factor, S = specific factor.

^*^ *p*<.05; ^**^ *p*<.01.

N = Non-clinical controls (N = 113), D = Depression (N = 30) B = Bipolar I Dx(N = 32).

### Common and specific factors related to the onset of bipolar disorder

With the diagnostic group as an independent variable and the 17 subscale scores as dependent variables, ANOVAs were conducted for each of the 17 subscales. The results, with post hoc analyses, are presented in Table 3. Results show that manic emotional response was the only specific factor related to the onset of BD. Common factors related to both MDD and BD were learning risky activities, reinforcing impulsivity, grandiose self-labeling, exposure to irritability, punishment of negative emotions, lack of social support, sleep problems, antidepressant problems, positive arousal to threat, a lack of social skills, short-term pleasure seeking, and a lack of problem solving skills. Twelve variables were identified as common factors.

## Discussion

BD is one of the most severe mental illnesses and is accompanied by reduced functioning for the individual. The lifetime prevalence of this disorder is 2% [16]. Initial onset generally occurs in late adolescence or early adulthood, severely disturbing both academic and occupational performance [17]. BD can result in severe maladjustment, although prognosis can be improved if pharmacotherapy and psychotherapy are provided simultaneously [18]. However, there are few evidence-based psychotherapy targeting its etiological factors. In order to support the development of this type of psychotherapy program, in this study, common and specific factors related to the onset of MDD and BD were explored.

According to psychological behaviorism theory, the etiological factors underlying BD are past psychosocial factors, current psychosocial factors, current transient organic factors, and basic behavioral repertoires. The main task in the development of the BDES was composing items not related to symptoms themselves, but rather to behavioral tendencies affecting the symptoms as etiological factors, especially in the case of basic behavior repertoires.

The results show that among past psychosocial factors, learning risky activities, reinforcing impulsivity, grandiose self-labeling, exposure to irritability, and punishment of negative emotions are common factors affecting the onset of both MDD and BD. This shows that many MDD and BD patients have learned to engage in risky activities, been reinforced for their impulsivity, been exposed family members’ irritability, and been punished for expressing negative emotions. Among current psychosocial factors, a lack of social support was identified as a common factor affecting both MDD and BD. Among the current transient organic factors, sleep disturbance and antidepressant problems were identified as common factors. In e-m BBRs, positive arousal to threat was a common factor. Positive arousal to threat, which is part of the e-m BBR, was identified as a common factor related to both MDD and BD. This sub-factor was hypothesized to be a specific factor because manic patients tend to manifest pleasure-seeking behaviors with positive arousal to anticipated ego-threatening results. However, this sub-factor too, has been identified as a common factor affecting MDD and BD.

A lack of social skills, which is part of the s-m BBR, was also identified as a common factor. When stressful events occur, patients with MDD or BD fail to seek appropriate social support, which aggravates their mood symptoms.

Short-term pleasure seeking and a lack of problem solving skills, in the l-c BBR, were identified as common factors. Short-term pleasure seeking was hypothesized to be a specific factor, but it was a common factor. This is because we could not develop items that were specifically related to typical manic symptoms. We tried to focus the items on BBRs as behavioral trends during periods of remission, not the symptoms themselves. For this reason, the items measuring short-term pleasure seeking resembled the failure of delayed gratification. In a follow-up study of BD patients who are in remission and do not manifest sexual promiscuity and compulsive consumption behaviors, the possibility that they ‘want to’ engage in this kind of short-term pleasure-seeking behaviors should be explored. As for the lack of problem solving skills, this seems to be a common factor underlying many mental disorders, not just MDD and BD.

It is notable that family members’ grandiose labeling proved to be a common factor. This result is consistent with the finding that excessive praise during childhood has adverse effects [19]. However, more research is needed to examine the difference between the effects of grandiose labeling and excessive praise. Not all individuals exposed such an atmosphere during childhood develop a mood disorder. Examining the details of this result, unlike for the other common factors, the BD group has higher scores on this subscale than the non-clinical control group, but there is no significant difference between the BD and MDD groups or between the MDD and non-clinical control groups. In a future study on a larger sample, if the difference between individuals with BD and MDD is significant, but that between the MDD and non-clinical control groups is not significant, this subscale can be considered as a specific factor.

The only specific factor was manic emotional response. The items measuring this subscale consist of statements about respondents’ experiences during remission, not during an active manic episode. This suggests that the tendency to be easily elated remains during the maintenance therapy period and that a cognitive intervention to treat this tendency can be employed during the period of remission, not just during manic episodes.

In summary, the specific factors identified in this study were manic emotional response and grandiose self-labeling. In addition, we identified several common factors between MDD and BD. These findings were not surprising, and were consistent with previous notions that MDD and BD are spectrum disorders. Another important contribution of this study was that we found thirteen factors associated with BD. In other words, although some of the features of BD may commonly be applicable to MDD, counselors working with BD can now develop a structured intervention program specifically designed for BD. Such an intervention program for BD must be designed to treat residual elating tendency, i.e., the manic emotional response, even though BD patients do not overtly exhibit symptoms of elation. Likewise, even though there is no evidence of BD patients showing grandiose ideation overtly, the therapist must treat their possible grandiose self-labeling. As a matter of fact, a CBT intervention program based on the findings of this research was developed and implemented with BD patients in Korea. In the treatment sessions related to low self-esteem and depressed moods, some of the BD patients expressed a kind of grandiose ideation as a rational thought to replace irrational and self-defeating thoughts, though they were not in an active manic episode. For instance, a patient shared with the therapist, “I’m the greatest scientist in the world. I’m the most beautiful woman in the world”. According to this finding, we must carefully scrutinize the rational thoughts that BD patients express in CBT sessions. The therapist must confirm whether there is a characteristic of grandiose ideation in the rational thought expressed by the BD patient to replace irrational thoughts. The other common factors identified in the present study can be used to develop a specific intervention for BD and MDD. For example, reinforcing impulsivity and short-term pleasure seeking subscales were found to be common factors in this study. Therefore, intervention to evaluate the long term effects of impulsive and pleasure seeking activities in BD and MDD patients can be effective in ameliorating the symptoms of BD and MDD. The main limitation of this study is related to the sample size. This study must be replicated in a larger sample. The low validity of the some subscales is also a limitation of this study. All subscales had acceptable validity on at least one of the validity indexes. However, the grandiose self-labeling subscale was not significantly correlated with the corresponding visual analog scale, and the euphoria-triggering experience and manipulative skill subscales were not significantly correlated with the diagnostic status variable. Thus, revision of these subscales should also be taken into consideration. Some of the common factors may be identified as specific factors after scale revision.

## Supporting Information

S1 Table **Bipolar Disorder Etiology Scale based on psychological behaviorism.**
(DOCX)

S2 Table **Subscales of BDES and their matched item numbers.**
(DOCX)

S3 Table **Visual Analog Scales for the hypotheses.**
(DOCX)
