# Supplementary material for: Validation of the Bipolar Disorder Etiology Scale Based on Psychological Behaviorism Theory and Factors Related to the Onset of Bipolar Disorder
Source: PLoS One. 2014 Dec 30;9(12):e116265. doi: 10.1371/journal.pone.0116265 (PMC4280146; doi:10.1371/journal.pone.0116265)
Supplement: S1 Table — Bipolar Disorder Etiology Scale based on psychological behaviorism. (DOCX) [file pone.0116265.s001.docx]

**Table S1. Bipolar Disorder Etiology Scale based on psychological behaviorism**

| Read the following statements about your experiences and thoughts, and then indicate how much you agree with them. | | | | | | |  |
| --- | --- | --- | --- | --- | --- | --- | --- |
| 1 = strongly disagree, 2 = disagree, 3 = slightly disagree, 4 = slightly agree, 5 = agree, 6 = strongly agree | | | | | | |  |
| 1. While growing up, at least one of my family members abused drugs. | 1 | 2 | 3 | 4 | 5 | 6 |  |
| 2. While growing up, at least one of my family members gambled. | 1 | 2 | 3 | 4 | 5 | 6 |  |
| 3. While growing up, at least one of my family members engaged in risky activities under the influence of alcohol. | 1 | 2 | 3 | 4 | 5 | 6 |  |
| 4. While growing up, at least one of my family members spent time in prison. | 1 | 2 | 3 | 4 | 5 | 6 |  |
| 5. While growing up, I learned risky activities from one of my family members. | 1 | 2 | 3 | 4 | 5 | 6 |  |
| 6. While growing up, at least one of my family members made fun of me when I would not take a risk and make a realistic plan. | 1 | 2 | 3 | 4 | 5 | 6 |  |
| 7. While growing up, at least one of my family members praised my risky activities. | 1 | 2 | 3 | 4 | 5 | 6 |  |
| 8. While growing up, none of my family members taught me to consider the consequences of risky behavior. | 1 | 2 | 3 | 4 | 5 | 6 |  |
| 9. While growing up, none of my family members taught me to make realistic plans for accomplishing goals. | 1 | 2 | 3 | 4 | 5 | 6 |  |
| 10. While growing up, at least one of my family members pressured me to be far more successful than most people. | 1 | 2 | 3 | 4 | 5 | 6 |  |
| 11. While growing up, at least one of my family members told me “You are great.” Therefore, I believed I was. | 1 | 2 | 3 | 4 | 5 | 6 |  |
| 12. When our family faced problems, one of my family members would ignore the realistic coping and solution. | 1 | 2 | 3 | 4 | 5 | 6 |  |
| 13. While growing up, at least one of my family members always seemed proud of what I had done. | 1 | 2 | 3 | 4 | 5 | 6 |  |
| 14. While growing up, it did not take much for me to feel like a success. | 1 | 2 | 3 | 4 | 5 | 6 |  |
| 15. While growing up, the smallest success with a little effort would make me feel very happy. | 1 | 2 | 3 | 4 | 5 | 6 |  |
| Read the following statements about your experiences and thoughts, and then indicate how much you agree with them. | | | | | | |  |
| 1 = strongly disagree, 2 = disagree, 3 = slightly disagree, 4 = slightly agree, 5 = agree, 6 = strongly agree | | | | | | |  |
| 16. Every short-term success, with no long-term consequences, makes me feel quite happy. | 1 | 2 | 3 | 4 | 5 | 6 |  |
| 17. I’ve enjoyed gambling and have won money at the gambling table. | 1 | 2 | 3 | 4 | 5 | 6 |  |
| 18. I can succeed at my job without working very hard. | 1 | 2 | 3 | 4 | 5 | 6 |  |
| 19. One day, I realized I am a person with special religious or spiritual abilities. | 1 | 2 | 3 | 4 | 5 | 6 |  |
| 20. Because others like me, I feel I am an important person. | 1 | 2 | 3 | 4 | 5 | 6 |  |
| 21. I have no social network to lead me in the right direction. | 1 | 2 | 3 | 4 | 5 | 6 |  |
| 22. I have few social relationships to share my fun. | 1 | 2 | 3 | 4 | 5 | 6 |  |
| 23. There is nobody around me to warn me not to choose the wrong path. | 1 | 2 | 3 | 4 | 5 | 6 |  |
| 24. I have had no role model to show me a stable lifestyle. | 1 | 2 | 3 | 4 | 5 | 6 |  |
| 25. I have had no mentor to be my role model. | 1 | 2 | 3 | 4 | 5 | 6 |  |
| 26. I receive encouragement to do risky things. | 1 | 2 | 3 | 4 | 5 | 6 |  |
| 27. My friends and family think I will be a very special person. | 1 | 2 | 3 | 4 | 5 | 6 |  |
| 28. People I know encourage me to take risks and have adventures. | 1 | 2 | 3 | 4 | 5 | 6 |  |
| 29. People I know encourage me to do things that are unconventional, but that makes me happy and I am interested as well. | 1 | 2 | 3 | 4 | 5 | 6 |  |
| 30. I am really a great person who changes the world. | 1 | 2 | 3 | 4 | 5 | 6 |  |
| Read the following statements about your experiences and thoughts, and then indicate how much you agree with them. | | | | | | |  |
| 1 = strongly disagree, 2 = disagree, 3 = slightly disagree, 4 = slightly agree, 5 = agree, 6 = strongly agree | | | | | | |  |
|  |  |  |  |  |  |  |  |
| 31. Sometimes, I do not sleep well. | 1 | 2 | 3 | 4 | 5 | 6 |  |
| 32. My sleep is irregular. | 1 | 2 | 3 | 4 | 5 | 6 |  |
| 33. Racing thoughts sometimes keep me from sleeping. | 1 | 2 | 3 | 4 | 5 | 6 |  |
| 34. I prefer to daydream rather than sleep. | 1 | 2 | 3 | 4 | 5 | 6 |  |
| 35. On holidays, I used to go to bed and wake up very late. | 1 | 2 | 3 | 4 | 5 | 6 |  |
| 36. I used to take antidepressant medication. | 1 | 2 | 3 | 4 | 5 | 6 |  |
| 37. Antidepressant medication used to make me feel quite happy. | 1 | 2 | 3 | 4 | 5 | 6 |  |
| 38. Because of the side effects, my doctor stopped antidepressant medication and prescribed another medication. | 1 | 2 | 3 | 4 | 5 | 6 |  |
| 39. When I was treated for depression, my diagnosis turned out to be wrong. | 1 | 2 | 3 | 4 | 5 | 6 |  |
| 40. When I was treated with antidepressant medication, I experienced an elated mood. | 1 | 2 | 3 | 4 | 5 | 6 |  |
| 41. While watching a movie, I tend to identify with the leader actor so much that I lose my sense of reality. | 1 | 2 | 3 | 4 | 5 | 6 |  |
| 42. My religious experiences are profound. | 1 | 2 | 3 | 4 | 5 | 6 |  |
| 43. When I read a myth or fantasy novel, I daydream that I became the hero of the story and I do great things. | 1 | 2 | 3 | 4 | 5 | 6 |  |
| 44. I usually identify with the hero in a movie or book. | 1 | 2 | 3 | 4 | 5 | 6 |  |
| 45. Someday, I will be the hero in the drama of my life. I will accomplish great things. | 1 | 2 | 3 | 4 | 5 | 6 |  |

| Read the following statements about your experiences and thoughts, and then indicate how much you agree with them. | | | | | | |  |
| --- | --- | --- | --- | --- | --- | --- | --- |
| 1 = strongly disagree, 2 = disagree, 3 = slightly disagree, 4 = slightly agree, 5 = agree, 6 = strongly agree | | | | | | |  |
| 46. I am not afraid to have multiple sex partners. | 1 | 2 | 3 | 4 | 5 | 6 |  |
| 47. There is no reason to worry about gambling large amounts of money. | 1 | 2 | 3 | 4 | 5 | 6 |  |
| 48. I do not feel threatened when I do something risky but pleasurable. | 1 | 2 | 3 | 4 | 5 | 6 |  |
| 49. I am less afraid of things like sky-diving and skin-scuba than most people seem to be. | 1 | 2 | 3 | 4 | 5 | 6 |  |
| 50. When I see someone bother a person who is weak, I don’t hesitate to help the weak. | 1 | 2 | 3 | 4 | 5 | 6 |  |
| 51. It is easy for me to attract sex partners. | 1 | 2 | 3 | 4 | 5 | 6 |  |
| 52. I am good at gambling. | 1 | 2 | 3 | 4 | 5 | 6 |  |
| 53. It is easy for me to do something risky. | 1 | 2 | 3 | 4 | 5 | 6 |  |
| 54. I have social skills to manipulate others. | 1 | 2 | 3 | 4 | 5 | 6 |  |
| 55. I have many credit cards I can use to buy things I need. | 1 | 2 | 3 | 4 | 5 | 6 |  |
| 56. When I am stressed out, I have few resources I can use to overcome the situation. | 1 | 2 | 3 | 4 | 5 | 6 |  |
| 57. When I am stressed out, I am not good at asking others for help. | 1 | 2 | 3 | 4 | 5 | 6 |  |
| 58. I rarely feel a need to apologize, even when I make a mistake. | 1 | 2 | 3 | 4 | 5 | 6 |  |
| 59. When I face difficult situations, it is hard for me to ask for help. | 1 | 2 | 3 | 4 | 5 | 6 |  |
| 60. If I’m having problems in my relationship, my functional level is easily affected. | 1 | 2 | 3 | 4 | 5 | 6 |  |
| Read the following statements about your experiences and thoughts, and then indicate how much you agree with them. | | | | | | |  |
| 1 = strongly disagree, 2 = disagree, 3 = slightly disagree, 4 = slightly agree, 5 = agree, 6 = strongly agree | | | | | | |  |
| 61. I would do anything to get pleasure right now. | 1 | 2 | 3 | 4 | 5 | 6 |  |
| 62. Accomplishing something valuable, after overcoming hardships, is nonsense. | 1 | 2 | 3 | 4 | 5 | 6 |  |
| 63. If I can get satisfied immediately, I don’t consider what happens next. | 1 | 2 | 3 | 4 | 5 | 6 |  |
| 64. I don’t understand someone who gives up pleasurable things today for a bright future. | 1 | 2 | 3 | 4 | 5 | 6 |  |
| 65. Nobody knows what tomorrow will bring, so it’s important to enjoy pleasure today. | 1 | 2 | 3 | 4 | 5 | 6 |  |
| 66. When I am sad, I can deny it and pretend to be doing fine. | 1 | 2 | 3 | 4 | 5 | 6 |  |
| 67. When sad things happen, I manage to get along well, without noticing my sad feelings. | 1 | 2 | 3 | 4 | 5 | 6 |  |
| 68. If I fail at something, I am even more confident that things will go better and be greater in the future. | 1 | 2 | 3 | 4 | 5 | 6 |  |
| 69. Because of my current status, people doesn’t know my true value. | 1 | 2 | 3 | 4 | 5 | 6 |  |
| 70. The fact that I will become a great figure in the future makes me ignore my current status. | 1 | 2 | 3 | 4 | 5 | 6 |  |
| 71. When I have a problem, I think deeply about it and cope with it in order to solve it. | 1 | 2 | 3 | 4 | 5 | 6 |  |
| 72. When I have a relationship problem, I contemplate it deeply and cope with it well. | 1 | 2 | 3 | 4 | 5 | 6 |  |
| 73. I have coping skills to solve the occupational problems. | 1 | 2 | 3 | 4 | 5 | 6 |  |
| 74. When I am misunderstood by someone, I have skills to make myself understood. | 1 | 2 | 3 | 4 | 5 | 6 |  |
| 75. When I am stressed out because of relationship problems at home or at work, I am good at contemplating and handling this kind of problems. | 1 | 2 | 3 | 4 | 5 | 6 |  |

| Read the following statements about your experiences and thoughts, and then indicate how much you agree with them. | | | | | | |  |
| --- | --- | --- | --- | --- | --- | --- | --- |
| 1 = strongly disagree, 2 = disagree, 3 = slightly disagree, 4 = slightly agree, 5 = agree, 6 = strongly agree | | | | | | |  |
| 76. While growing up, at least one of my family members had angry outbursts. | 1 | 2 | 3 | 4 | 5 | 6 |  |
| 77. While growing up, at least one of my family members verbally abused other family members. | 1 | 2 | 3 | 4 | 5 | 6 |  |
| 78. While growing up, at least one of my family members used to show irritability. | 1 | 2 | 3 | 4 | 5 | 6 |  |
| 79. While growing up, at least one of my family members used to lose his or her temper. | 1 | 2 | 3 | 4 | 5 | 6 |  |
| 80. While growing up, at least one of my family members used to show hostility toward other members. | 1 | 2 | 3 | 4 | 5 | 6 |  |
| 81. While growing up, at least one of my family members told me that expressing negative feelings was not good. | 1 | 2 | 3 | 4 | 5 | 6 |  |
| 82. While growing up, at least one of my family members punished me because I expressed anger. | 1 | 2 | 3 | 4 | 5 | 6 |  |
| 83. While growing up, at least one of my family members rejected my expression of negative feelings. | 1 | 2 | 3 | 4 | 5 | 6 |  |
| 84. While growing up, at least one of my family members didn’t accept me expressing negative feelings. | 1 | 2 | 3 | 4 | 5 | 6 |  |
| 85. While growing up, at least one of my family members tried to forcefully alter the way I expressed my negative feelings. | 1 | 2 | 3 | 4 | 5 | 6 |  |
