# Supplementary material for: Validation of the Bipolar Disorder Etiology Scale Based on Psychological Behaviorism Theory and Factors Related to the Onset of Bipolar Disorder
Source: PLoS One. 2014 Dec 30;9(12):e116265. doi: 10.1371/journal.pone.0116265 (PMC4280146; doi:10.1371/journal.pone.0116265)
Supplement: S2 Table — Subscales of BDES and their matched item numbers. (DOCX) [file pone.0116265.s002.docx]

**Table S2. Subscales of BDES and their matched item numbers**

| **Subscale** | **Item No.** | **Subscale** | **Item No.** | **Subscale** | **Item No.** |
| --- | --- | --- | --- | --- | --- |
| 1. Learning risky activities | 1–5 | 7. Lack of social support | 21–25 | 13. Manipulative Skill | 51–55 |
| 2. Reinforcing impulsivity | 6–10 | 8. Reinforcement of elevated emotions | 26–30 | 14. Lack of social skills | 56–60 |
| 3. Grandiose self- labeling | 11–15 | 9. Sleep problems | 31–35 | 15. Short-term pleasure seeking | 61–65 |
| *4. Exposure to irritability | *76–80 | 10. Antidepressant problems | 36–40 | 16. Denial of negative emotions | 66–70 |
| *5. Punishment of negative emotions | *81–85 | 11. Manic emotional response | 41–45 | 17. Lack of problem-solving skills | 71–75 |
| 6. Euphoria-triggering experience | 16–20 | 12. Positive arousal to threat | 46–50 |  |  |

*Subscales 4 and 5 were added to the original model. Thus, their item numbers are higher than those of the other subscales.
