# Supplementary material for: Validation of the Bipolar Disorder Etiology Scale Based on Psychological Behaviorism Theory and Factors Related to the Onset of Bipolar Disorder
Source: PLoS One. 2014 Dec 30;9(12):e116265. doi: 10.1371/journal.pone.0116265 (PMC4280146; doi:10.1371/journal.pone.0116265)
Supplement: S3 Table — Visual Analog Scales for the hypotheses. (DOCX) [file pone.0116265.s003.docx]

**Table S3. Visual Analog Scales for the hypotheses**

Read the following statements about your experiences and thoughts, and then indicate how much you agree with them.

1 = strongly disagree / 10 = strongly agree

| 1. While growing up, I learned to indulge in risky activities from my family members. | **1** | **2** | **3** | **4** | **5** | **6** | **7** | **8** | **9** | **10** |
| --- | --- | --- | --- | --- | --- | --- | --- | --- | --- | --- |
| 2. While growing up, my family members punished realistic future plans, and reinforced impulsivity and denial of consequences. | **1** | **2** | **3** | **4** | **5** | **6** | **7** | **8** | **9** | **10** |
| 3. While growing up, I learned to use grandiose self-labeling and denial as ways to cope with stress as well as to enhance feelings of elation and confidence in response to superficial successes. | **1** | **2** | **3** | **4** | **5** | **6** | **7** | **8** | **9** | **10** |
| 4. While growing up, I have observed irritability in my family members. | **1** | **2** | **3** | **4** | **5** | **6** | **7** | **8** | **9** | **10** |
| 5. While growing up, I used to be punished for expressing negative emotions including anger. | **1** | **2** | **3** | **4** | **5** | **6** | **7** | **8** | **9** | **10** |
| 6. Recently, there have been superficially pleasant events that evoked abnormal euphoric emotion in me, such as winning money during gambling. | **1** | **2** | **3** | **4** | **5** | **6** | **7** | **8** | **9** | **10** |
| 7. I have little social support that reinforces euthymic emotions and safe behaviors, and to warns me appropriately. | **1** | **2** | **3** | **4** | **5** | **6** | **7** | **8** | **9** | **10** |
| 8. People near me provided reinforcement for elevated emotions, grandiose self-labeling, and impulsive and sensual activities. | **1** | **2** | **3** | **4** | **5** | **6** | **7** | **8** | **9** | **10** |
| 9. I have experienced sleep deprivation or sleep disturbances. | **1** | **2** | **3** | **4** | **5** | **6** | **7** | **8** | **9** | **10** |
| 10. I have ever taken antidepressants carelessly. | **1** | **2** | **3** | **4** | **5** | **6** | **7** | **8** | **9** | **10** |
| 11. I have excessive bipolar emotional responses to certain stimulus situations. | **1** | **2** | **3** | **4** | **5** | **6** | **7** | **8** | **9** | **10** |
| 12. I tend to have problems because I respond with positive emotional arousal to situations that others find threatening or anxiety provoking. | **1** | **2** | **3** | **4** | **5** | **6** | **7** | **8** | **9** | **10** |
| 13. I have above-average skills for engaging in risky types of activities, which produce short-term reinforcement and long-term punishment. These may include persuasive conversational techniques or social manipulation. | **1** | **2** | **3** | **4** | **5** | **6** | **7** | **8** | **9** | **10** |
| 14. I have a deficit in the social skills required to get adequate social support. | **1** | **2** | **3** | **4** | **5** | **6** | **7** | **8** | **9** | **10** |
| 15. If I can get short-term benefits, I pay little attention to long-term negative consequences. | **1** | **2** | **3** | **4** | **5** | **6** | **7** | **8** | **9** | **10** |
| 16. I tried to forget or suppress negative emotions or thoughts. | **1** | **2** | **3** | **4** | **5** | **6** | **7** | **8** | **9** | **10** |
| 17. I have poor cognitive problem-solving skills. | **1** | **2** | **3** | **4** | **5** | **6** | **7** | **8** | **9** | **10** |
